# Three-Year Results from the Venovo Venous Stent Study for the Treatment of Iliac and Femoral Vein Obstruction

**DOI:** 10.1007/s00270-021-02975-2

**Published:** 2021-09-20

**Authors:** Michael D. Dake, Gerard O’Sullivan, Nicolas W. Shammas, Michael Lichtenberg, Bibombe P. Mwipatayi, Richard A. Settlage, for the VERNACULAR Trial Investigators

**Affiliations:** 1 University of Arizona Health Sciences, Health Sciences Innovation Building, 9Th Floor SVP Suite,1670 E. Drachman Street, P.O. Box 210216, Tucson, AZ 85721-0216 USA; 2 University Hospital Galway, Galway, Ireland; 3 Midwest Cardiovascular Research Foundation, Davenport, IA USA; 4 Arnsberg Vascular Clinic, Arnsberg, Germany; 5 University of Western Australia Royal Perth Hospital, Perth, Australia; 6 Becton Dickinson Peripheral Interventions, Tempe, AZ USA

**Keywords:** Iliofemoral venous obstruction, Iliac and femoral vein occlusive disease, Percutaneous endovenous stent, Post-thrombotic syndrome, Non-thrombotic iliac vein lesion

## Abstract

**Purpose:**

To assess safety and patency of the Venovo venous stent for the treatment of iliofemoral vein obstruction.

**Materials and Methods:**

Twenty-two international centers enrolled 170 patients in the VERNACULAR study (93 post-thrombotic syndrome; 77 non-thrombotic iliac vein lesions). Primary outcome measures were major adverse events at 30 days and 12-month primary patency (freedom from target vessel revascularization, thrombotic occlusion, or stenosis > 50%). Secondary outcomes included the Venous Clinical Severity Score Pain Assessment and Chronic Venous Quality-of-Life Questionnaire assessments (hypothesis tested). Secondary observations included primary patency, target vessel and lesion revascularization (TVR/TLR), and assessment of stent integrity through 36 months.

**Results:**

Freedom from major adverse events through 30 days was 93.5%, statistically higher than a pre-specified performance goal of 89% (*p* = 0.032) while primary patency at 12 months was 88.6%, also statistically higher than a performance goal of 74% (*p* < 0.0001). Mean quality-of-life measures were statistically improved compared to baseline values at 12 months (*p* < 0.0001). Primary patency at 36 months was 84% (Kaplan–Meier analysis) while freedom from TVR/TLR was 88.1%. There was no stent embolization/migration, and no core laboratory assessed stent fractures reported through 36 months. Six deaths were reported; none adjudicated as device or procedure related.

**Conclusion:**

The Venovo venous stent was successfully deployed in obstructive iliofemoral vein lesions and met the pre-specified primary outcome measures through 12 months. At 3 years, primary patency was 84%, reintervention rates were low, standardized quality-of-life and pain measures improved from baseline, and there was no stent migration or fractures.

**Level of Evidence:**

Level 2—prospective, multicenter, controlled clinical study without a concurrent control or randomization. Pre-specified endpoints were hypothesis-tested to performance goals derived from peer-reviewed clinical literature.

**Registration clinicaltrials.gov:**

Unique Identifier NCT02655887.

## Introduction

Iliofemoral venous obstruction, caused by anatomic vein compression or deep vein thrombosis (DVT), can lead to severe venous insufficiency and a reduction in quality-of-life. Exercise, compression therapy, anticoagulation with thrombolysis, catheter-directed thrombolysis, or pharmaco-mechanical thrombectomy do not address the underlying causes of venous obstruction and have provided mixed therapeutic results [1–3]. Surgery has been used to bypass occlusions, but stent use has largely replaced surgery as the interventional procedure of choice [4]. Bare metal stents designed for arterial use have been used to treat peripheral venous obstructions, yet few have been systematically studied in the venous system [5–7]. Dedicated venous stents for iliofemoral venous outflow obstruction have emerged over the past few years as alternatives to conservative therapy, surgery, and the use of arterial stents. Observational single-center studies using the Venovo venous stent have reported good early patency and low complication rates [8–10]. The current prospective, multicenter study was designed and powered to evaluate major adverse events and primary patency of this dedicated, nitinol self-expanding, open-cell venous stent for iliofemoral obstructions through 12 months with secondary observations through 3 years.

## Methods and Materials

### Study Design

VERNACULAR was a prospective, multicenter study of the Venovo venous stent used to treat iliac and femoral vein thrombotic obstruction or venous compression. Between June 2016 and May 2017, 170 patients were treated at 22 centers in the USA, Europe, and Australia. Investigators followed a protocol approved by their institutional review board or ethics committee, patients provided written informed consent to participate in the study, and study procedures were conducted in accordance with the Declaration of Helsinki, good clinical practice, and other applicable healthcare regulations and privacy laws. A clinical events committee (CEC) provided independent oversight of patient safety, and a medical monitor reviewed adverse event trends. The Yale Cardiovascular Research Group (New Haven, CT) independently analyzed X-rays and venographic images while VasCore, the Vascular Ultrasound Core Laboratory (Massachusetts General Hospital, Boston, MA), reviewed duplex-ultrasound (DUS) images. VERNACULAR was sponsored by Bard/Becton, Dickinson and registered on clinicaltrials.gov (Unique Identifier: NCT02655887) prior to patient enrollment.

Eligible patients had symptomatic, non-malignant venous outflow obstruction in the iliac or femoral veins (≥ 50% by contrast venography) with a clinical–etiologic–anatomic–pathophysiologic clinical score (CEAP “C” score) ≥ 3 or a venous clinical severity score (VCSS) pain component ≥ 2. Exclusion criteria included venous obstruction that extended into the inferior vena cava or below the level of the lesser trochanter, prior stent placement in the target vessel, or an iliac or femoral vein unsuitable for treatment with available device sizes.

### Outcome Measures

The primary outcome measures included freedom from major adverse events (MAEs) through 30 days and 12-month primary patency, both compared to performance goals (PGs) derived from published clinical literature. MAEs included target vessel revascularization (TVR), device- or procedure-related DVT, target-limb major amputation, symptomatic pulmonary embolism, vascular injury requiring intervention, device migration, or death. Primary patency was defined as freedom from TVR, thrombotic occlusion, or stenosis greater than 50% measured by DUS and reviewed by the core laboratory. Secondary outcomes included quality-of-life (QoL) measures evaluated with the pain component of the VCSS and the Chronic Venous Quality-of-Life Questionnaire (CIVIQ-20 Global Index). QoL assessments were hypothesis-tested at 12 months to mean baseline values. Additional secondary observations included technical success, defined as successful deployment of the stent to the intended location with adequate lesion coverage and procedural success, technical success with no major adverse events between index procedure and discharge. Primary patency, TVR and target lesion revascularization (TLR; both core laboratory evaluated), CEAP classification scores, and stent integrity (i.e., migration and fracture) were reported through 3 years.

### Baseline Patient and Lesion Characteristics

**Fig. 1:**
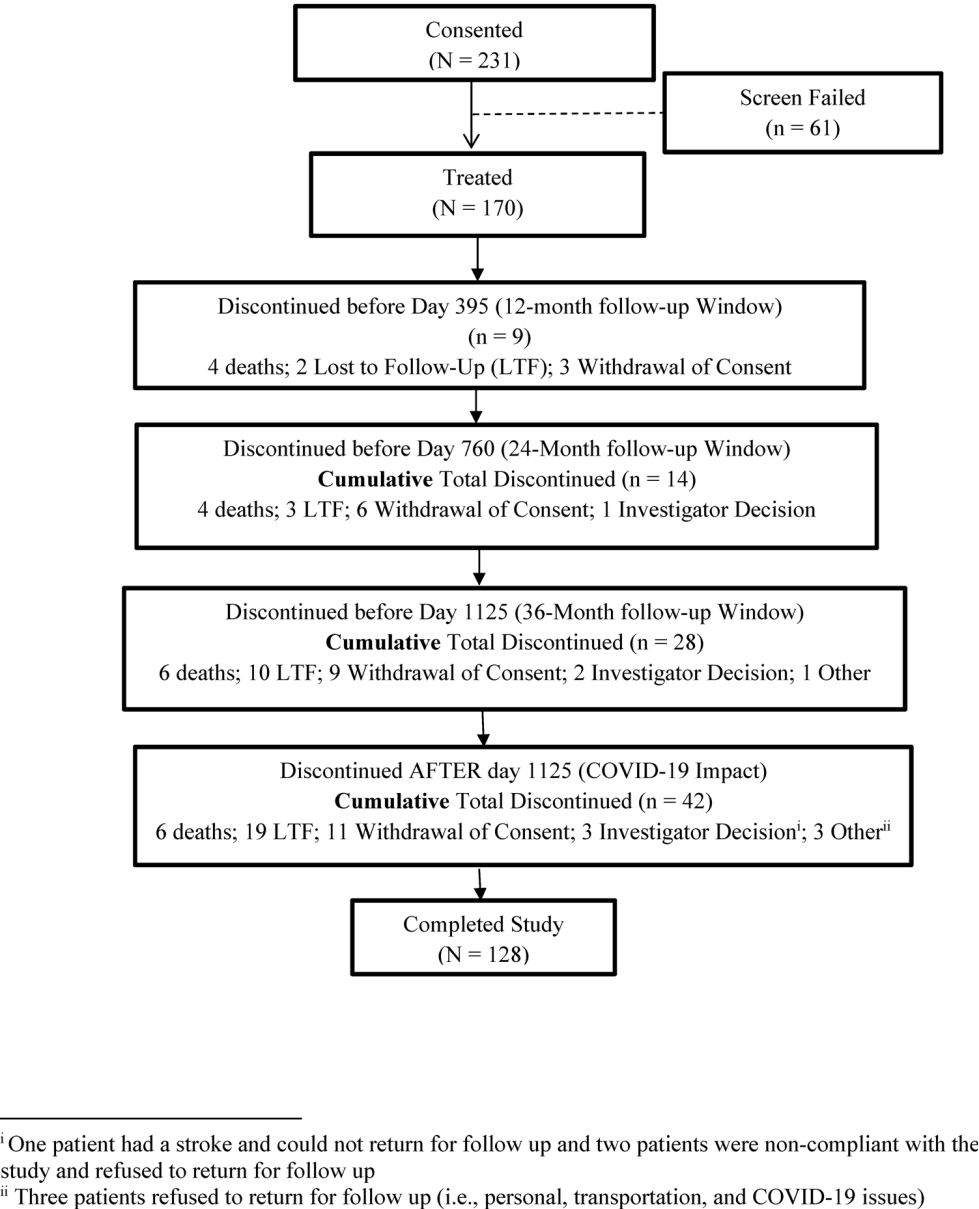
Summary of the final disposition of patients in the VERNACULAR Study. Two hundred thirty-one patients were enrolled, and 170 were treated with the Venovo venous stent. One hundred twenty-eight patients completed the study while 42 patients either withdrew consent or were withdrawn by an investigator (14), were lost to follow-up (19), died (6), or discontinued participation for other reasons (3). Due to the COVID-19 pandemic, the final 36-month follow-up window was extended beyond 1125 days to allow patients to complete the study

The treated population consisted of 170 patients who consented to participate in the study and received a stent (Fig. 1) and included 93 patients with post-thrombotic syndrome (PTS) and 77 patients with symptomatic non-thrombotic iliac vein lesions (NIVL). Baseline patient demographics and medical histories are summarized in Table 1. At baseline, 235 lesions were evaluated by the venographic core laboratory. Patients could present with more than one lesion in multiple veins; 94.5% of patients had lesions in the common iliac vein, 40.5% in the external iliac vein, and 9.2% in the common femoral vein. The mean total lesion length per treated limb was 67.8 ± 39.7 mm, and the mean pre-procedure percent diameter stenosis was 75.7 ± 17.0% (Table 2).

**Table 1:** Baseline patient demographics, medical history, and additional procedures

Patient demographics	PTS subgroup *N* = 93	NIVL subgroup *N* = 77	All treated *N* = 170
Age (years), X ± SD	49.8 ± 15.0	55.0 ± 15.4	52.1 ± 15.3
Male/Female, % / %	45.2/54.8	27.3/72.7	37.1/62.9
BMI, kg/m^2^ ± SD	28.6 ± 6.4	29.1 ± 7.7	28.8 ± 7.0
*Race, % (n)*			
Caucasian	89.2 (83)	94.8 (73)	91.8 (156)
African American	7.7 (7)	2.6 (2)	5.3 (9)
Asian	3.2 (3)	1.3 (1)	2.4 (4)
*Medical history, % (n)*			
Varicose Veins	76.3 (71)	80.5 (2)	78.2 (133)
May–Thurner Syndrome	37.6 (35)	87.0 (67)	60.0 (102)
Deep Vein Thrombosis	98.9 (92)	0 (0)	54.1 (92)
Cigarette Smoking	30.1 (28)	39.0 (30)	34.1 (58)
Hypertension	29.0 (27)	36.4 (28)	32.4 (55)
Dyslipidemia	21.5 (20)	35.1 (27)	27.6 (47)
Respiratory Disorder	14.0 (13)	9.1 (7)	11.8 (20)
Diabetes	5.4 (5)	16.9 (13)	10.6 (18)
Peripheral Arterial Disease	6.5 (6)	15.6 (12)	10.6 (18)
Gastrointestinal Disease	11.8 (11)	9.1 (7)	10.6 (18)
Coronary Artery Disease	6.5 (6)	11.7 (9)	8.8 (15)
Coronary Intervention	6.5 (6)	10.4 (8)	8.2 (14)
Venous Valve Disease	7.5 (7)	6.5 (5)	7.1 (12)
Inferior Vena Cava Filter	10.8 (10)	0 (0)	5.9 (10)
*Additional procedures*^1^*, % (n)*			
Previous Procedure^2^	20.4 (19)	13.0 (10)	17.1 (29)
30-Day Planned Procedure^3^	9.7 (9)	2.6 (2)	6.5 (11)

^1^Some patients had multiple procedures

^2^Procedure within 1 year of the study; most peripheral vascular interventions were for vena cava filter placement (33%), thrombectomy (16%), or venous procedures (e.g., venoplasty, ablation, or lysis)

^3^Planned peripheral endovascular or surgical intervention 30 days before or after the study procedure

**Table 2:** Baseline lesion characteristics and stent details

Lesion characteristics^1^	PTS subgroup *N* = 89	NIVL subgroup *N* = 74	All treated *N* = 163^2^
*Lesion Location*^3^*, % (n/N)*			
Common Iliac Vein	92.1 (82/89)	10.8 (8/74)	94.5 (154/163)
External Iliac Vein	58.4 (52/89)	18.9 (14/74)	40.5 (66/163)
Common Femoral Vein	14.6 (13/89)	2.7 (2/74)	9.2 (15/163)
Lesion Length, mm, μ ± SD	80.5 ± 42.8	55.2 ± 32.0	67.8 ± 39.7
Length Range, mm	18.1–199.7	22.3–183.4	18.1–199.7
Vessel Diameter, mm, μ ± SD	16.0 ± 2.7	17.4 ± 3.3	16.7 ± 3.0
*Diameter Stenosis, %* ± *SD*			
Baseline	81.0 ± 18.4	69.3 ± 12.6	75.7 ± 17.0
After Pre-dilation	49.9 ± 21.1	40.6 ± 28.2	47.8 ± 22.9
Final	16.1 ± 7.0	12.1 ± 5.3	14.3 ± 6.5
*Stent Details*^4^			
Diameter, mm, μ ± SD	15.4 ± 2.1	16.6 + 2.0	15.9 ± 2.1
Length, mm, μ + SD	100.1 ± 33.2	83.0 ± 26.3	93.5 ± 31.7
Stents Placed, N	134	85	219
Single Stent, % (n)	69.4 (93)	90.6 (77)	77.6 (170)
Distal Overlap, % (n)	20.1 (27)	4.7 (4)	14.2 (31)
Proximal Overlap, % (n)	10.4 (14)	4.7 (4)	8.2 (18)
Pre-dilation, % (n)	87.1 (81)	66.2 (51)	77.6 (132)
Post-dilation, % (n)	92.5 (86)	88.3 (68)	90.6 (154)
Technical Success, % (n/N)	100 (93/93)	100 (77/77)	100 (170/170)
Procedural Success, % (n/N)	97.8 (91/93)	100 (77/77)	98.8 (168/170)^5^

^1^Quantitative Vascular Analysis by the Venographic Core Laboratory

^2^163 patients in the ITT cohort had images that could be evaluated by the core laboratory

^3^Some patients had lesions in multiple locations

^4^219 stents were used in 170 patients

^5^Two patients in the PTS subgroup experienced a TVR for stent thrombosis prior to discharge

### Study Procedures and Follow-Up

A comprehensive clinical history, physical examination, QoL assessment, and diagnostic venogram were performed prior to treatment to confirm study eligibility. Patients received prophylactic low-molecular-weight heparin prior to the procedure, 3000–5000 Units of unfractionated heparin during the procedure, and appropriate antiplatelet or anticoagulation medications post-procedure (e.g., acetylsalicylic acid, clopidogrel, rivaroxaban, apixaban) based on physician and institution standard-of-care guidelines. The Venovo venous stent (Bard/Becton, Dickinson and Company, Tempe, Arizona, USA) was used according to the device instructions for use. Stent diameters ranged from 10 to 20 mm (2 mm increments) and lengths from 40 to 160 mm (20 mm increments), the stent ends were flared approximately 3 mm larger than nominal stent diameter to provide fixation and prevent migration, and three radiopaque tantalum markers on each end of the stent enhanced visibility to aid in placement. Stents were introduced through an 8–10 F sheath depending on device size. Up to two stents per patient were allowed per protocol with a recommended overlap of 10 mm. Venograms were completed pre-deployment, at stent deployment, and at procedure completion and were sent to the venographic core laboratory for analysis. Follow-up visits at 1, 6, 12, 24, and 36 months included physical examination, target-limb assessment, documentation of reintervention or adverse events, assessment of changes in QoL, and DUS imaging. Anterior–posterior (A–P) radiographs were taken at 12, 24, and 36 months to evaluate possible stent fractures (analyzed by the Yale Core Laboratory).

### Statistical Analysis

The treated sample size of 170 patients provided an overall power of > 85% for the pre-specified outcome measures. Freedom from MAEs was achieved if the one-sided p value was less than 0.05 or the lower limit of the two-sided 90% confidence limit was greater than a literature-derived PG of 89% (exact binomial method). MAEs reported in 19 peer-reviewed articles (> 2400 patients) were used to derive a mean overall freedom from MAEs of 99% [11–29]. The PG was set at a 10% non-inferiority margin below the literature-derived value at 30 days. Similarly, primary patency was achieved if the one-sided p value was less than 0.05 or the lower limit of the two-sided 90% confidence limit was greater than a PG of 74% (weighted Z-statistics). The patency PG was derived from published data on stent treatment of lower-limb venous obstructions; primary patency data from fifteen NIVL studies (1149 limbs) [11, 13, 15, 23, 25, 27, 28, 30–37] and fourteen PTS studies (908 limbs) [11, 12, 14, 19–22, 24, 26, 28, 29, 38–40] were weighted and used to derive an overall mean primary patency rate (84%); the PG was set at a 10% non-inferiority margin below the weighted literature-derived mean at 12 months. The VCSS pain score and CIVIQ-20 assessment at 12 months were hypothesis-tested to baseline values (Hochberg method for controlling multiple comparisons); the reduction in pain and discomfort was deemed statistically significant if the two-sided p value (paired *t* test) was less than 0.05.

Additional secondary outcomes were reported using Kaplan–Meier (K–M) estimates and descriptive statistics through 3 years. Descriptive statistics included categorical variables presented as percentages and frequency counts, and continuous variables that included means ± standard deviation (SD) and confidence intervals (CIs). Secondary outcomes beyond the hypothesis-tested measures at 12 months were observational.

## Results

Two hundred nineteen (219) stents were used in 170 patients; one device was used in 77.6% of cases (Table 2). The mean stent length was 93.5 ± 31.7 mm (range 40–160 mm), and the mean stent diameter was 15.9 ± 2.1 mm (range 10–20 mm). Technical success was achieved in all cases (100%) while procedural success was 98.8% (168/170); two PTS patients experienced a TVR for stent thrombosis prior to discharge. The final mean percent diameter stenosis after stent placement was 14.3 ± 6.5%.

### Post-procedure Follow-up and Outcomes

**Fig. 2:**
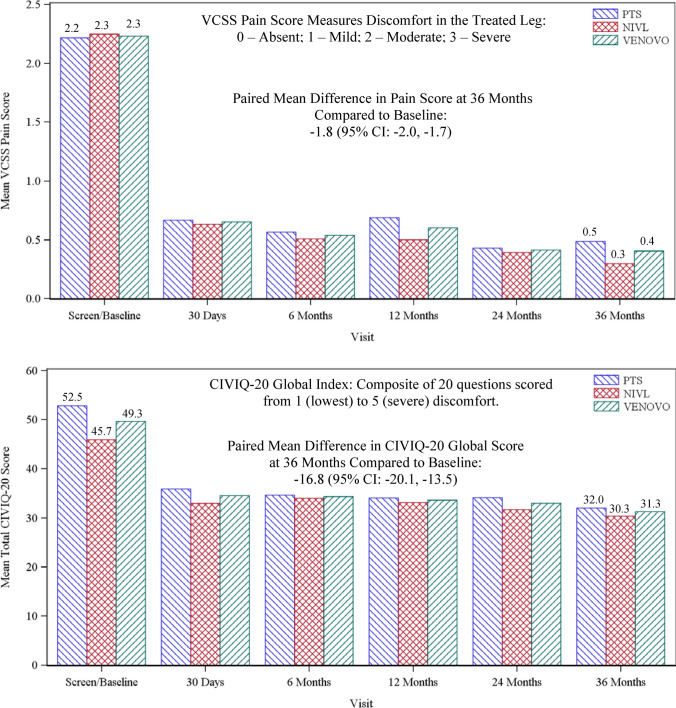
Quality-of-life summary bar graphs through 36 months. The paired mean improvement in the VCSS pain score at 36 months was -1.8 [95% CI: −2.0, −1.7] and the paired mean improvement in CIVIQ-20 global index score was −16.8 [95% CI: −20.1, −13.5] both compared to baseline values

One hundred twenty-eight patients completed the study at 3 years (Fig. 1). Primary and secondary outcome measures are summarized in Table 3. Freedom from MAEs at 30 days was 93.5% [90% CI: 89.5%, 96.3%], statistically higher than the literature-derived PG of 89% (one-sided exact binomial test; *p* value = 0.03). Analysis of patient subgroups demonstrated a numerical difference, but not a statistically significant difference in MAEs between the PTS subgroup (88.2%) and the NIVL subgroup (100%; *p* = 0.94 logistic regression with subgroup as fixed effect). Twelve-month primary patency was 88.6% [90% CI: 82.8%, 94.4%], also statistically higher than the literature-derived PG of 74% (one-sided exact binomial test; *p* value < 0.0001). This was a weighted mean based on the distribution between the mean primary patency for the PTS and NIVL subgroups. Analysis of patient subgroups demonstrated a statistically higher primary patency rate for the NIVL subgroup (96.9%) compared to the PTS subgroup (81.3%; *p* = 0.01; type III test by logistic regression with subgroup as fixed effect). The VCSS pain score and the CIVIQ-20 global index were secondary QoL endpoints hypothesis-tested at 12 months to baseline values. The mean improvement in the VCSS pain score was −1.7 [95% CI: −1.8, −1.5] at 12 months (two-sided paired *t* test; *p* < 0.0001; Fig. 2A) and the mean improvement in the CIVIQ-20 global index score was −15.7 [95% CI: −18.4, −13.0] (two-sided paired *t* test: *p* < 0.0001; Fig. 2B), representing statistically significant improvements in overall pain and patient comfort from baseline.

**Table 3:** Primary results, hypothesis-tested secondary outcomes, and secondary observations

Primary outcome measures	PTS subgroup *N* = 93	NIVL subgroup *N* = 77	All treated *N* = 170	*p* value [90% CI]
Primary Patency^1^, % (n//N)	81.7 (67/82)	97.1 (66/68)	88.6^2^	< 0.0001^3^ [82.8, 94.4]
Freedom from MAEs^4^, % (n/N)	88.2 (82/93)	100 (77/77)	93.5	0.032^5^ [89.5, 96.3]

Secondary outcome measures (hypothesis tested)				*p* value
*VCSS Pain Score*^6^				
Baseline	2.2^7^ [2.1, 2.4]	2.3 [2.1, 2.4]	2.3 [2.2, 2.4]	
12 Months	0.7 [0.5, 0.9]	0.5 [0.3, 0.7]	0.6 [0.5, 0.7]	
Change from Baseline	−1.5 [−1.8, −1.3]	−1.8 [−2.0, −1.6]	−1.7 [−1.8, −1.5]	< 0.0001^8^
*CIVIQ-20 Score*^9^				
Baseline	52.5^10^ [48.6, 56.4]	45.7 [41.8, 49.5]	49.3 [46.5, 52.0]	
12 Months	34.0 [30.5, 37.6]	33.1 [29.3, 36.9]	33.6 [31.0, 36.2]	
Change from Baseline	−18.5 [−22.2, −14.7]	−12.6 [−16.5, −8.7]	−15.7 [−18.4, −13.0]	< 0.0001^11^

Secondary observations				[95% CI]
*CEAP Score, mean* ± *SD*				
Baseline	3.4 ± 1.0	3.5 ± 0.8	3.5 ± 0.9	
36 Months	2.1 ± 1.6	2.0 ± 1.5	2.1 ± 1.6	
Change from Baseline	−1.3 [−1.7, −1.0]	−1.5 [−1.9, −1.2]	−1.4^12^ [−1.7, −1.2]	
*Freedom from TVR/TLR*				
12 Months, % (n/N)	87.6 (78/89)	98.6 (73/74)	92.6 (151/163)	[87.5, 96.1]
24 Months, % (n/N)	82.8 (72/87)	97.3 (72/74)	89.4 (144/161)	[83.6, 93.7]
36 Months, % (n/N)	80.5 (66/82)	97.1 (67/69)	88.1 (133/151)	[81.8, 92.8]
Primary Patency^13^, % (n/N)				[90% CI]
24 Months	75.6 (62/82)	95.4 (62/65)	84.4 (124/147)	[78.6, 89.1]
36 Months	70.0 (49/70)	93.6 (44/47)	79.5 (93/117)	[72.4, 85.4]
Stent Migration^14^, % (n/N)	0 (0/93)	0 (0/77)	0 (0/170)	
*Stent Fracture*^15^*, % (n/N)*				
12 Months	0 (0/72)	0 (0/65)	0 (0/137)^16^	
24 Months	0 (0/65)	0 (0/63)	0 (0/128)	
36 Months	0 (0/53)	0 (0/45)	0 (0/98)	

^1^Primary patency was defined as freedom from TVR, thrombotic occlusion, or stenosis greater than 50% measured by duplex ultrasonography (DUS) and reviewed by the DUS core laboratory

^2^Weighted mean based on the patency rates of PTS and NIVL subgroups

^3^90% CI and one-sided p value is from the weighted Z-statistics and the combined patency rate was tested against the performance goal (PG) (74%)

^4^MAEs included target vessel revascularization (TVR), device- or procedure-related deep vein thrombosis (DVT), target-limb major amputation, symptomatic pulmonary embolism, vascular injury requiring either surgical or endovascular intervention, device migration, or death

^5^The p value is computed compared with performance goal of 89%. The 90% confidence interval is calculated using the exact binomial method

^6^Pain component of the venous clinical severity score (VCSS) hypothesis-tested against baseline values at 12 months

^7^Mean and 95% CI are presented based on normal assumption

^8^The p value is calculated from a two-sided paired *t* test

^9^The Chronic Venous Quality-of-Life Questionnaire (CIVIQ-20) score hypothesis-tested against baseline values at 12 months

^10^Mean and 95% CI are presented based on normal assumption

^11^The p value is calculated from a two-sided paired *t* test

^12^Mean and 95% CI are presented based on normal assumption

^13^Unweighted proportional values with 90% CI estimated by exact binomial method

^14^CEC adjudicated stent embolism/migration through 3 years

^15^Based on AP and lateral X-rays for each evaluated stent analyzed by the core laboratory

^16^Number of stents that had images available and readable for review

**Fig. 3:**
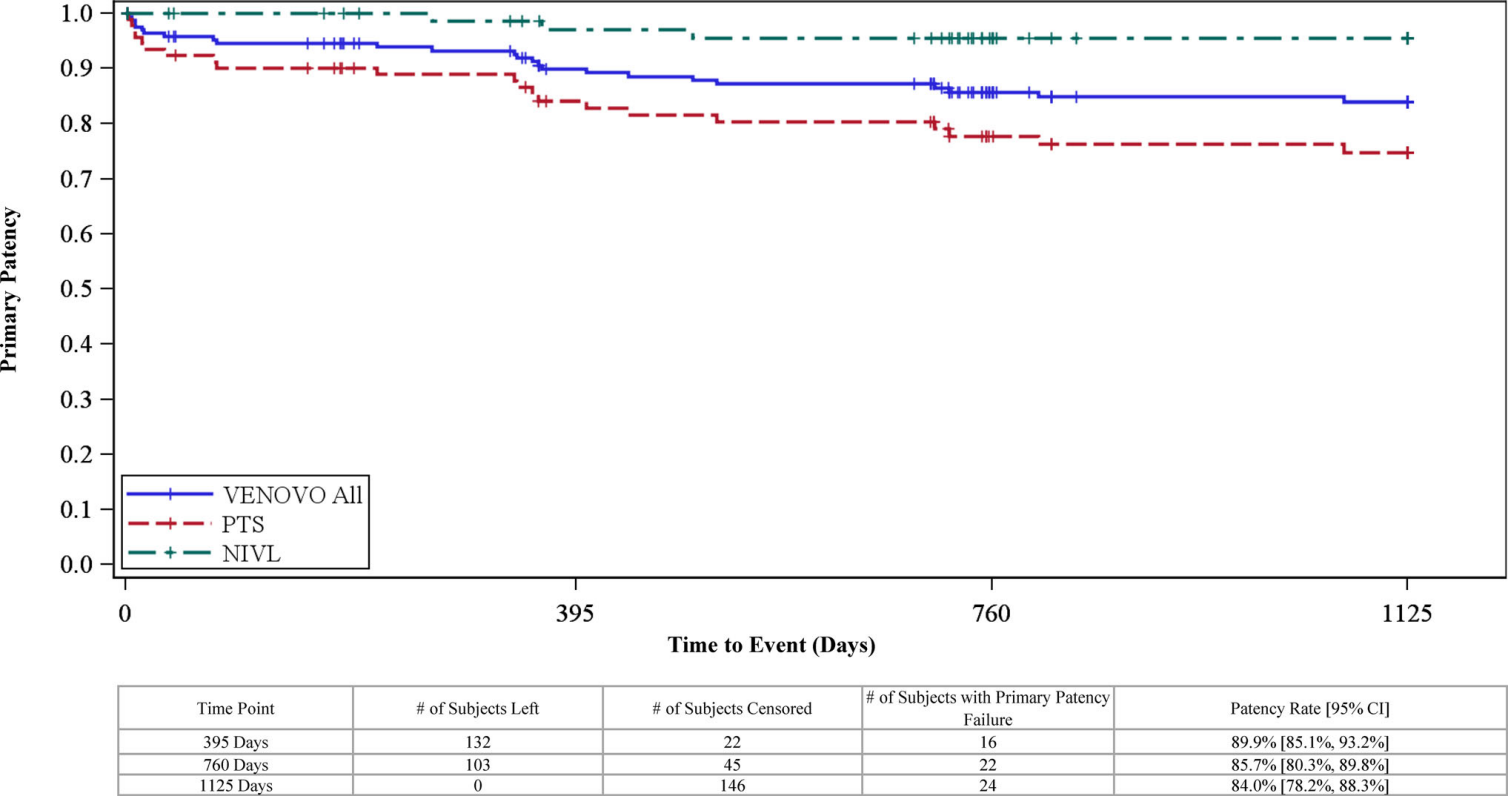
Kaplan–Meier curve of primary patency through 3 years. The estimated primary patency rate at 1125 days, the end of the 3-year follow-up window, was 84.0% [95% CI: 78.2%, 88.3%]

Supportive analyses and secondary observations without formal hypothesis testing were conducted through 3 years. The unweighted primary patency rate (binary analysis) was 79.5% [90% CI 72.4%, 85.4%] while the K–M estimate of primary patency, accounting for patients censored (e.g., lost to follow-up) in the binary analysis, was 84.0 ± 3.0% (1125 days: Fig. 3). The mean improvement from baseline in the VCSS pain score was −1.8 [95% CI: −2.0, −1.7], CIVIQ-20 global index was −16.8 [95% CI: −20.1, −13.5], and CEAP “C” score was −1.4 [95% CI: −1.7, −1.2]. Freedom from TVR/TLR (binary) was 88.1%. No stent embolization or migration was reported, and A−P and lateral radiographs at 12, 24, and 36 months, reviewed by the X-ray core laboratory, revealed no stent fractures.

Six deaths were reported through 3 years. In three cases, the investigator listed a specific cause of death (i.e., one case of rectal cancer and two cases of myocardial infarction). In the remaining three cases, the specific cause of death was not listed; post-mortem summaries, however, indicated the apparent causes as recurrent cancer, complications of liver cirrhosis, and age-related natural causes. The CEC adjudicated that no deaths were related to the stent.

## Discussion

Contemporary clinical practice has established stenting of iliofemoral venous obstruction as a durable therapy with relatively few complications. Stents have been used most often in acute cases with severe symptoms and in chronic DVT patients with symptoms of moderate to severe PTS; most were designed for arterial use, so the guidance was appropriately prudent and meant to discourage overuse of arterial stents in cases without evidence-based justification [41, 42]. Four self-expanding nitinol stents, specifically designed to meet the challenges of venous obstructive disease, have now been FDA-approved and address the long-held realizations that venous lesions are fundamentally different than the pathological disease that typically affects arteries; that vessel compliance, wall composition, and blood flow are distinctly dissimilar between arteries and veins; and that the basic dimensions (e.g., diameter, wall thickness) of iliac arteries and veins are disparate.

One- and two-year data from the current trial and the three other venous-specific stent trials—VIRTUS (Vici Venous stent), VIVO (Zilver Vena), and ABRE (Abre venous stent)—have been presented or published [43–45]. The 3-year results of the VERNACULAR trial, however, are the longest-term follow-up data published on a venous-specific stent. Primary patency was superior to a literature-based performance goal at 1 year (*p* < 0.0001) and was sustained at 3 years (79.5%) with a relative drop in the patency rate of only 9% from 1 to 3 years. The Kaplan–Meier estimate of primary patency was 84% while freedom from TVR and TLR was 88.1% at 3 years.

It is difficult to compare results between the venous stent trials because of differences in patient demographics, anatomic characteristics as well as specific differences in protocol-defined inclusion criteria and outcome measures. There are also differences in stent designs, open cell (Venovo, Zilver Vena, Abre) versus closed cell (Vici); the former typically providing greater device flexibility while closed-cell stents accentuate resistance to external compression [46]. The Venovo stent has some distinct features such as 3 mm flared stent ends designed for anchoring and the largest venous stent diameter range (10–20 mm). Given the mean diameter of treated veins in multiple trials was > 15 mm, and 13% of patients in the VIRTUS trial were excluded because the vein diameter was < 12 mm or > 16 mm, the large diameter range may prove useful [43].

Although not directly comparable, observations from the four trials can provide insight on the performance of venous stents. First, patient demographics across trials were similar, composed predominantly of women (range 63% to 70%), with a mean age ranging from 51 to 54 years, and with lesions overwhelmingly affecting the left leg (up to 90%). Total occlusions were present in 26% to 32% of cases with a baseline mean diameter vein stenosis of 63–78%. Second, there appeared to be no clear differences in safety endpoints observed through 30 days between the studies based on similar composite metrics. Freedom from MAEs at 30 days in the VERNACULAR trial was 93.5%, statistically higher compared to the safety performance goal. Third, primary patency rates were clustered from 84 to 90% at 1 year. Patency outcomes for the NIVL subgroups ranged from 96 to 99%, and as anticipated, the patency outcomes for the PTS subgroups were uniformly lower (VERNACULAR 81.7%; VIRTUS 79.8%, ABRE 79.8%, and Vici Venous Stent Trial 59%). At 3 years, the primary patency rate for the VERNACULAR trial was 79.5% (93.6% and 70%, respectively, for the NIVL and PTS subgroups). Fourth, paralleling the patency outcomes, TVR-free rates through the first 24 months were consistent across the dedicated venous stent studies (e.g., VERNACULAR 89.4%, VIRTUS 88.7%) while the rate in the VERNACULAR trial remained consistent at 3 years (88.1%). Fifth, there was no stent embolization or migration and no stent fractures observed in the VERNACULAR trial through 3 years; migration (0–1%) and fractures (0–4%) were also rare in the other trials at 1 and 2 years. Finally, lower VCSS scores and improved quality-of-life results (EQ-5D Index, Chronic Venous Quality-of-Life Questionnaire-CIVIQ-20 Global Index) were observed through all follow-up time points compared to pre-intervention assessments. At 3 years in the VERNACULAR trial, the mean improvement from baseline in the VCSS pain score was −1.8 and in the CIVIQ-20 score was −16.8. Similar QoL improvements were observed in the other dedicated venous stent trials at earlier time points, but clinical outcome studies comparing arterial stents and dedicated venous stents for management of iliofemoral venous disease have not been performed.

Limitations of the current trial included the single-arm design that used historical rather than concurrent, randomized controls. The trial was prospective, multicentered, and had pre-specified endpoints powered to detect differences compared to performance goals; there can be selection bias, however, in the choice of historical studies. The case mix, trial design, and study methodology may be different than the studies used to determine the PGs, and the use of other studies could have yielded different results. The study was not designed to assess clinical success compared to non-venous stents. It is also possible that the Venovo stent performed differently than venous stents used in other trials. A comparative, randomized study powered to determine differences between stents would be needed within the same demographic population. Forty-two patients discontinued prior to the end of the study resulting in an overall follow-up rate of 75.3%. The COVID-19 pandemic impacted the final follow-up at 3 years, and an attrition of approximately 25% could have impacted the results. The primary outcome measures and secondary quality-of-life parameters at 12 months were statistically powered and hypothesis-tested, but additional secondary analyses out to 3 years were observational only.

Recent data from dedicated venous stent trials for iliac and femoral vein occlusive disease provide interventionalists with clear outcomes that can be anticipated through 1 year following stent placement. Three-year results from the VERNACULAR trial using the Venovo venous stent are the longest-term follow-up data from a controlled multicenter trial. Primary patency and stent integrity were sustained at 2 and 3 years, reintervention rates were low, and patient quality-of-life improved, providing physicians and patients with important predictability and confidence in the durability of the earlier outcomes.
